# The silent crisis of child abuse in the COVID‐19 pandemic: A scoping review

**DOI:** 10.1002/hsr2.790

**Published:** 2022-08-17

**Authors:** Zahra Karbasi, Reza Safdari, Parisa Eslami

**Affiliations:** ^1^ Department of Health Information Sciences, Faculty of Management and Medical Information Sciences Kerman University of Medical Sciences Kerman Iran; ^2^ Department of Health Information Management, School of Allied Medical Sciences Tehran University of Medical Sciences Tehran Iran

**Keywords:** child abuse, child maltreatment, COVID‐19, violence

## Abstract

**Background and Aims:**

The global outbreak of COVID‐19 has become an international concern. The lives of children are severely affected by COVID‐19 pandemic. There is evidence of a pandemic impact on violence against children. This scoping review study aimed to investigate the effects of the COVID‐19 pandemic on child abuse.

**Methods:**

We searched PubMed, Scopus, and Web of Science databases to retrieve related studies. Regarding the recent incident of COVID‐19, the articles were reviewed from 2019 to June 1, 2021. The terms Child abuse and COVID‐19 were used in the precise search technique of each database. The search techniques were created to work with any scientific database that used the keywords given.

**Results:**

In the initial search of scientific databases, 568 articles were retrieved. After applying the inclusion and exclusion criteria during the screening process, 16 papers were included in the scoping review. Twelve articles have mentioned the increase of physical, psychological, and neglect types of abuse. However, sexual violence has not been reported in any of the articles. Four articles reported a reduction in the incidence of child abuse.

**Conclusion:**

During the COVID‐19 pandemic, a crisis occurred in the form of an upsurge in violence toward children, since limits made to diminish the virus, in general, increased the danger to children. Numerous factors such as stress, poverty, financial situation, history of violence, school closures, and lack of contact with support organizations contribute to this phenomenon. Social action and support needed is the right of every child in need in this critical situation.

## 1 INTRODUCTION

The first human case of coronavirus disease 2019 (COVID‐19) was reported in December 2019.^1^, ^2^ The global outbreak of COVID‐19 has become an international concern.^3^ It is spreading rapidly, and this disease has affected millions of people worldwide.^4^ COVID‐19 may be readily transferred by respiratory droplets and direct touch. Numerous COVID‐19 individuals have symptoms including fever, cough, severe headache, loss of taste or smell, and exhaustion.^5^ More than 524 million instances of COVID‐19 have been verified worldwide as of May 25, 2022, and more than 6 million fatalities have been documented.^6^ COVID‐19 pandemic as a new disease has affected many children which caused them to develop inflammatory syndromes, although the severity of symptoms is lower in children than in adults.^7^ The disease is asymptomatic^8^ in children or with mild symptoms.^9^

The lives of children are severely affected by the COVID‐19 pandemic, which has led to negative consequences for them. There is evidence of a pandemic impact on violence against children. It is often not exposed, and many children do not receive the support they deserve.^10^ Besides, the situation and house quarantines aggravate this issue and may lead to negligence and abuse.^11^ Child abuse is, unfortunately, a worldwide issue with long‐term harmful implications. Child abuse affects children under the age of 18 and may be perpetrated by their parents, caregivers, or strangers. Physical, emotional, sexual, and neglect are all examples of abuse. Each of these leads to damage to the health, survival, and growth of the child. About 40,000 deaths occur each year in terms of child abuse.^12^

The World Health Organization (WHO) warns of an increase in child abuse considering the spread of COVID‐19 and home quarantine. Because children have less awareness and access to move out of the home and obtain aid, the problem of child maltreatment becomes particularly concerning in this circumstance.^13^ According to studies, parents are the most common perpetrators of violence toward children.^14^, ^15^ During the COVID‐19 pandemic, the families suffered various economic pressures, restrictions, and lack of access to support services.^16^ The pandemic created a situation for people which increased mental disorders and the severity of symptoms. Anxiety was heightened by restrictions on remaining at home and adhering to health regimens. Younger patients are negatively affected by pandemic stress. During this time, the symptoms and severity of mental diseases, particularly obsessive‐compulsive disorder in youngsters, rose.^17^

COVID‐19 caused many children to spend time at home with their parents,^18^ and violence against children often occurs in the home and family.^19^ Parents and caregivers who lost their jobs as a result of the pandemic are much more negligent of their children. Children are more likely to be abused at home as a result of these changes and extreme stress, and the bad consequences may last a long time.^20^ Job loss is linked to child maltreatment, according to the findings of systematic review research conducted in the United Kingdom. Sexual abuse was shown to be less associated with work and money, but physical abuse was found to be more related to income and employment.^21^ During the COVID‐19 pandemic, there was an upsurge in child maltreatment, according to evidence.^22^, ^23^, ^24^ The COVID‐19 pandemic has created problems for families and children that communities were not prepared to deal with it.^25^ Regarding the present global environment and the COVID‐19 dilemma, it is critical to assess the position of children who are most at danger of violence. This scoping review study aimed to investigate the effects of the COVID‐19 pandemic on child abuse. Therefore, various forms of violence against children during the COVID‐19 pandemic were examined.

## 2 METHODS

We followed the five‐step methodological framework of Arksey and O'Malley.^26^ This includes (1) identifying questions of research, (2) identifying relevant studies, (3) selecting studies, (4) extracting data, (5) summarizing, and describing results. Providing the nature and features of research in the field of interest and short duration are among the strengths of this method. It also provides results in a short and accessible way to consumers and makes it possible to identify gaps.

According to Arksey and O'Malley 's framework, our scoping review questions were

(1) Has the COVID‐19 pandemic affected the incidence of child abuse?
(2) Which type of child abuse has the COVID‐19 pandemic increased?
(3) What were the risk factors for child abuse during the pandemic?

We searched PubMed, Scopus, and Web of Science databases to retrieve related studies. Regarding the recent incident of COVID‐19, the articles were reviewed from 2019 to June 1, 2021. The search techniques were created to work with any scientific database that used the keywords given. The terms “Child abuse” and “COVID‐19” were used in the precise search techniques of each database. Table 1 shows the specifics of the search technique.

**Table 1:** Details of search strategy by databases

Data base	Search strategy
PubMed	((((COVID‐19 [MeSH Terms]) OR (COVID‐19 virus disease)) OR (coronavirus disease‐19)) OR (COVID‐19 pandemic)) AND ((((child abuse[MeSH Terms]) OR (child mistreatment)) OR (child maltreatment)) OR (child neglect))
Scopus	(TITLE‐ABS‐KEY (COVID‐19) OR TITLE‐ABS‐KEY (COVID‐19 AND virus AND disease) OR TITLE‐ABS‐KEY (coronavirus AND disease‐19) OR TITLE‐ABS‐KEY (COVID‐19 AND pandemic) AND TITLE‐ABS‐KEY (child AND abuse) OR TITLE‐ABS‐KEY (child AND mistreatment) OR TITLE‐ABS‐KEY (child AND maltreatment) OR TITLE‐ABS‐KEY (child AND neglect))
Web of Science	TS = ((“COVID 19”OR “COVID‐19 Virus Disease” OR “Coronavirus Disease‐19” OR “COVID‐19 Pandemic”) AND (“Child abuse” OR “Child Mistreatment” OR “Child Maltreatment” OR “Child Neglect”))

### 2.1 Inclusion criteria

To select the articles, the following inclusion criteria were defined: (1) Articles focused on the impact of COVID‐19 on the incidence of child abuse and (2) Included studies were limited to those written in the English language.

### 2.2 Exclusion criteria

Articles with at least one of these exclusion criteria were excluded: (1) The title, abstract, or full text of the article was not related to the effect of COVID‐19 on child abuse, (2) articles not written in English, (3) articles published in the form of an abstract, reviews and editorial papers, reports, working papers, technical reports, books, letters to editors, short briefs, and commentaries, and (4) full text was not available.

### 2.3 Data extraction

**Figure 1:**
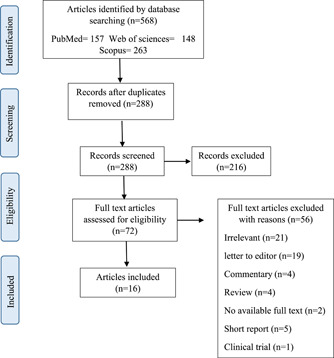
The PRISMA diagram for the selection related papers

The present scoping review article was performed based on the Preferred Reporting Items for Systematic Reviews and Meta‐Analyses (PRISMA) protocol. The PRISMA flow chart of the article selection process is presented in Figure 1. To manage citations, retrieved articles were entered into the Endnote X7 library, and duplicates were removed. To discover possibly relevant papers, the two writers individually participated in each article review process (screening, eligibility, and inclusion). According to the inclusion and exclusion criteria, they assessed the titles and abstracts of all non‐duplicated papers. Then, if the articles were unclear or differed upon, they screened the whole text of the articles and addressed the issues with the third reviewer through dialog or consultation. The entire text of relevant papers was then retrieved and examined in accordance with the final inclusion criteria. After the final review of the articles, the following data were extracted from the included studies: authors, year, country, journal, increased violence, risk factors, measures, type of study, sample size, and results.

**Table 2:** Summary of reviewed articles

Author	Year	Country	Journal	Increased violence				Risk factors	Measures	Type of study	Sample size	**Results**
				Physical	Emotional	Sexual	Neglect					
Abokresha et al.^27^	2021	Egypt	*Journal of the Egyptian Public Health Association*	*	*	_	_	Low parental education, low income, and large number of children	Online survey	Cross‐sectional study	Parents (*n* = 364)	The majority of the parents reported violence against their children during the COVID‐19 pandemic
Barboza et al.^28^	2020	United States	*Child Abuse and Neglect*	_	_	_	_	Poverty, absence from school, financial situation and housing, and labor force participation	Negative binomial regression	Cross‐sectional study	_	Reports of child abuse decreased during the outbreak of COVID‐19 pandemic
Brown et al.^29^	2020	United States	*Child Abuse and Neglect*	_	_	_	_	Financial assistance, parental stress, anxiety, and depression	Online survey	Cross‐sectional study	Parents (*n* = 183)	Findings indicate that parental stress and anxiety in the COVID‐19 pandemic increase violence against children
Calvano et al.^30^	2021	Germany	*European Child & Adolescent Psychiatry*	_	*	_	_	History of parental violence, age of parents, parental stress, job losses, and financial status	Online survey and telephone survey	Cross‐sectional study	Parents (*n* = 1024)	The results of this study showed that rates of child abuse and neglect increased during the COVID‐19 pandemic
Chong et al.^31^	2020	Singapore	*BMC Pediatrics*	*	_	_	_	_	Analysis of electronic health records data	Retrospective study	_	This study showed an increase in the proportion of complaints and diagnoses related to child abuse during the COVID‐19 pandemic
Chung et al.^32^	2020	Singapore	*Journal of Family Violence*	_	_	_	_	Parental stress, and job loss	Online survey	Cross‐ sectional study	Parents (*n* = 258)	According to the results of the study, COVID‐19 crisis is effective in increasing parental stress, which in turn is effective in increasing child abuse
Kovler et al.^33^	2020	United States	*Child Abuse and Neglect*	*	_	_	_	__	Analysis of patients data	Retrospective study	Patients <15 years of age (*n* = 257)	The results showed that physical injuries increased during the COVID‐19 pandemic
Lawson et al.^34^	2020	United States	*Child Abuse and Neglect*	*	*	_	_	Job loss, history of parental violence, and symptoms of depression	Online survey	Cross‐ sectional study	Parents (*n* = 342)	The results showed that parents who lost their jobs during the COVID‐19 pandemic were more likely to abuse their children physically and mentally
Lee et al.^23^	2020	United States	*Journal of Family Violence*	*	*	‐	*	Social isolation, job loss, and depressive symptoms	Online survey	Cross‐ sectional study	Parents (*n* = 283)	The results showed that parental social isolation during the COVID‐19 pandemic era increased neglect, physical and psychological abuse of children
Rapoport et al.^35^	2020	United States	*Child Abuse and Neglect*	_	_	_	_	_	Analysis of child abuse reporting data	Longitudinal study	_	Findings from the study show a significant reduction in the report of child abuse during COVID‐19
Rodriguez et al.^36^	2020	United States	*Child Maltreatment*	*	*	_	*	Job loss, food insecurity, loneliness, and parental stress	Online national survey	First study: cross‐sectional study Second study: prospective longitudinal study	First study: parents (*n* = 405) Second study: parents (*n* = 106)	The results of both studies showed that parents understood their conflict with their children during the pandemic
Sanford et al.^37^	2021	United States	*Journal of Pediatric Surgery*	_	_	_	_	_	Analysis of patients data	Retrospective study	_	The results showed that the proportion of traumas decreased during COVID‐19
Shah et al.^38^	2021	India	*Journal of Neurosciences in Rural Practice*	*	*	_	_	_	Online survey and text message based intervention	Cross‐ sectional study	Parents (*n* = 80)	According to the results, shouting at the child, verbal violence, and punishment of the child increased in the pandemic
Sharma et al.^22^	2021	United States	*Child Abuse and Neglect*	_	*	_	*	_	Analysis of electronic medical records data	Retrospective study	Children (*n* = 776)	According to the results of the study, the incidence of child abuse has increased during the COVID‐19 pandemic
Whelan et al.^39^	2020	United States	*Child Abuse and Neglect*	_	_	_	_	_	Analysis of child abuse report data	Longitudinal study	_	The results of the study show a decreasing trend in cases of child abuse. However, the risk factors for violence have increased
Wong et al.^24^	2021	China	*International Journal of Environmental Research and Public Health*	*	_	_	_	Job loss, and lower income	Online survey	Cross‐sectional study	Parents (*n* = 600)	The results showed that child abuse increased during the COVID‐19 pandemic and increased the risk of severe physical assault

## 3 RESULTS

### 3.1 Study selection

In the initial search of scientific databases, 568 articles were retrieved: Web of Science = 148, PubMed = 157, and Scopus = 263 articles. First, 280 articles were deleted in terms of duplication. Articles were first studied based on the title and abstracts. Then, 216 articles were discarded due to irrelevance. After applying the inclusion and exclusion criteria during the screening process, 16 papers were included in the scoping review.

### 3.2 Study characteristics

A summary of general specifications for the studies included in Table 2 is provided. We categorized the findings based on the distribution characteristics of the articles as follows:

## 4 Characteristics of the articles

### 4.1 Country

Based on the findings of this article, 10 articles were conducted in the United States and two articles were performed in Singapore. The other four studies were conducted in China, Egypt, Germany, and India.

### 4.2 Year of publication

As we can see, among the 16 articles published, 10 articles were published in 2020, and 6 articles were published in 2021.

### 4.3 Publisher journal

Based on the findings, 43.75% of the total articles were published in the journal of *Child Abuse and Neglect*. The remaining articles were published in other journals.

### 4.4 Risk factors

In 25% of the articles, parental stress was mentioned as the main factor in the incidence of violence against children. Household employment status, job loss and income, food poverty, the number of children in the family, the age of the children and parents, and a history of violence were all risk factors. Table 2 lists all the risk variables for violence against children during the COVID‐19 pandemic.

### 4.5 Measures

The majority of articles (56.25%) were done through online review and 43.75% through related data analysis.

### 4.6 Type of study

According to the findings, 62.5% of the articles were cross‐sectional and the rest were longitudinal or retrospective.

### 4.7 Sample size

Details of the sample size are given in Table 2.

### 4.8 Results

The findings revealed that eight of the 16 publications retrieved demonstrated an increase in the prevalence of child physical abuse during the COVID‐19 outbreak. Seven articles published during the crisis found an increase in the frequency of psychological maltreatment. In three publications, there was an increase in neglect, but no increase in sexual abuse. In some articles, the simultaneous increase of several types of abuse was mentioned.

## 5 DISCUSSION

There have been many challenges in the world since the outbreak of COVID‐19. One of the concerns raised during this period has been violence against children. A thorough understanding of the effects of the COVID‐19 pandemic on children and the risk factors for violence against them is a research requirement. During the COVID‐19 pandemic, we looked into the degree of child maltreatment. In addition, we looked at the risk variables for child violence over this time period. Twelve publications in this scoping review noted a rise in physical, psychological, and neglect kinds of abuse, according to the results of the review of studies.^22^, ^23^, ^24^, ^27^, ^29^, ^30^, ^31^, ^32^, ^33^, ^34^, ^36^, ^38^ However, sexual violence has not been reported in any of the articles. Four articles reported a reduction in the incidence of child abuse,^28^, ^35^, ^37^, ^39^ which could be in terms of the lack of reporting of the incident by individuals during the pandemic. The reporting of abuse was hindered during this time of quarantine owing to a lack of contact and access by children to centers, schools, or relatives and acquaintances.^40^, ^41^

According to a study by Seddighi et al., violence such as physical violence, neglect, and abuse increases after many emergencies and disasters, which is much more common than usual. In this area, child caretakers, particularly parents, have been highlighted as a key source of violence toward children.^15^ Children are more likely to be abused following natural catastrophes such as earthquakes, hurricanes,^42^ and wars, according to research.^43^, ^44^ Reports indicate that children are more likely to be abused in pandemics, such as Ebola^45^, ^46^ and the risk of domestic violence increases in terms of the pressures on families resulting from the crisis.^45^

According to the results of Campbell, decreased reports of child abuse during the pandemic are due to fewer opportunities to identify cases.^11^ According to the WHO, children are less likely to obtain assistance since they are unable to leave the house, and their young age makes them unaware of available resources.^8^ Caron et al. stated that the lack of reporting and screening of incidents during the lockdown has reduced the actual number of child abuse cases.^40^ This period doubles the concern because child victims are less able to get help from others.^47^

WHO has reported a 10%–50% increase in domestic violence helpline calls.^13^ Physical and mental abuse were the most prevalent forms of violence during the COVID‐19 pandemic, according to the results of this research. According to a report in the United States, the number of child abuse hospital visits fell during COVID‐19, while the number of child abuse hospitalizations rose compared to the previous year.^48^ In pandemics, studies have shown that there is a higher risk of violence.^49^, ^50^

Based on our findings, there was no evidence of sexual violence against children during the pandemic. This could be in terms of the child or witnesses not reporting this type of violence. Research in Kenya has shown that the pattern of sexual violence against children has changed during the COVID‐19 pandemic. Furthermore, younger children in private households were more likely to be mistreated.^51^ As a result, all public and private organizations should promote awareness about domestic violence to handle this situation.^52^

Regarding the complexity of child abuse, this crisis is affected by several factors that overlap.^53^ Various factors affect the occurrence of this violence. The results of our article showed that financial status and a job loss of parents, parental stress, gender and age of the child, parental anxiety and depression, and number of children in the family are the most important factors in increasing abuse against children during the COVID‐19 crisis. Evidence shows that most families in natural disasters are affected by socioeconomic pressures and even psychological pressures, and such families are more likely to commit violence against children.^44^, ^54^, ^55^ Quarantine and the ensuing limitations placed a lot of strain on families, which led to a rise in domestic violence against children, according to the findings of this research. Given that families and main caregivers are the most common perpetrators of violence against children in crises, more intervention is required.^15^

According to the Australian Institute of Health and Welfare, the factors such as financial problems, poor mental health, and housing concerns have increased child abuse during COVID‐19.^56^ Increased stress in interpersonal relationships and a history of mental disorders can all be identified as motivators of violence.^57^ Evidence suggests that COVID‐19 increases risk factors for abuse such as family and financial stress, and social constraints, but it is still unclear how much it contributes to increased violence.^58^

Based on the results discussed in this article, it is impossible to say that COVID‐19 causes child abuse, because quarantine, limitations, and risk factors may all contribute to violence. It is vital to remember that certain types of violence, such as sexual assault, may grow during a pandemic but are difficult to report owing to children's lack of communication outlets. Therefore, the management of this event requires special attention to various factors. The number of cases of child abuse may, in fact, be much higher than the number of reported cases, while due to the lack of reports of violence for various reasons, we will see a decrease in the trend of child abuse and even continue silently during the pandemic. This disrupts the monitoring and follow‐up of violence against children.

## 6 LIMITATION

The lack of extensive studies on violence against children during the pandemic was one of the limitations of this study. Therefore, the lack of reports of a decrease or increase in violence and the type of violence in some articles made it difficult to investigate the crisis.

## 7 CONCLUSION

COVID‐19 pandemic crisis itself, created a new crisis in many societies. Although child abuse often goes unreported, we must believe that the crisis has intensified in recent months.^10^ During the COVID‐19 pandemic, a crisis occurred in the form of an upsurge in violence toward children, since limits made to diminish the virus in general increased the danger to children. Numerous factors such as stress, poverty, and financial situation, history of violence, school closures, and lack of contact with support organizations contribute to this phenomenon. Social action and support needed is the right of every child in need in this critical situation.

## 8 RECOMMENDATION

Furthermore, urgent intervention measures are needed in this area to defend the rights of children via the necessary strategies and provide safe conditions for them during similar crises. To prevent violence, governments and support organizations should review child protection services during emergencies to ensure that necessary support is provided via improved communication. Managing preventive behaviors and informing the community, especially families, is one of the main models of response and risk management that choosing the best solution with long‐term consequences is very necessary to eliminate the risk. At the same time, it is important to note that supportive forms should be most compatible with the crisis period and beyond. We suggest that other studies examine other aspects of child maltreatment during and after the pandemic.

## AUTHOR CONTRIBUTIONS

*The conception and design of the study*: Zahra Karbasi and Parisa Eslami. *Methodology*: Zahra Karbasi, Reza Safdari, and Parisa Eslami. *Acquisition of data*: Zahra Karbasi and Parisa Eslami. *Writing—original draft preparation*: Zahra Karbasi and Parisa Eslami. *Writing—review and editing*: Zahra Karbasi, Reza Safdari, and Parisa Eslami. *Validation*: Zahra Karbasi, Reza Safdari, and Parisa Eslami. All authors have read and approved the final version of the manuscript. Zahra Karbasi had full access to all of the data in this study and takes complete responsibility for the integrity of the data and the accuracy of the data analysis.

## CONFLICT OF INTEREST

The authors declare no conflict of interest.

## TRANSPARENCY STATEMENT

Zahra Karbasi affirms that this manuscript is an honest, accurate, and transparent account of the study being reported; that no important aspects of the study have been omitted; and that any discrepancies from the study as planned (and, if relevant, registered) have been explained.

## Data Availability

The authors confirm that the data supporting the findings of this study are available within the article [and/or] its supplementary materials.
